# The utility of automatic segmentation of kidney MRI in chronic kidney disease using a 3D convolutional neural network

**DOI:** 10.1038/s41598-023-44539-z

**Published:** 2023-10-13

**Authors:** Kaiji Inoue, Yuki Hara, Keita Nagawa, Masahiro Koyama, Hirokazu Shimizu, Koichiro Matsuura, Masao Takahashi, Iichiro Osawa, Tsutomu Inoue, Hirokazu Okada, Masahiro Ishikawa, Naoki Kobayashi, Eito Kozawa

**Affiliations:** 1 Department of Radiology, Saitama Medical University, 38 Morohongou, Moroyama-machi, Iruma-gun, Saitama, Japan; 2 Department of Nephrology, Saitama Medical University, 38 Morohongou, Moroyama-machi, Iruma-gun, Saitama, Japan; 3 Department of Electronic Engineering and Computer Science, Faculty of Engineering, Kindai University Hiroshima Campus, 1 Takaya Umenobe, Higashi-Hiroshima City, Hiroshima Japan; 4 School of Biomedical Engineering, Faculty of Health and Medical Care, Saitama Medical University, 38 Morohongou, Moroyama-machi, Iruma-gun, Saitama, Japan

**Keywords:** Nephrology, Image processing

## Abstract

We developed a 3D convolutional neural network (CNN)-based automatic kidney segmentation method for patients with chronic kidney disease (CKD) using MRI Dixon-based T1-weighted in-phase (IP)/opposed-phase (OP)/water-only (WO) images. The dataset comprised 100 participants with renal dysfunction (RD; eGFR < 45 mL/min/1.73 m^2^) and 70 without (non-RD; eGFR ≥ 45 mL/min/1.73 m^2^). The model was applied to the right, left, and both kidneys; it was first evaluated on the non-RD group data and subsequently on the combined data of the RD and non-RD groups. For bilateral kidney segmentation of the non-RD group, the best performance was obtained when using IP image, with a Dice score of 0.902 ± 0.034, average surface distance of 1.46 ± 0.75 mm, and a difference of − 27 ± 21 mL between ground-truth and automatically computed volume. Slightly worse results were obtained for the combined data of the RD and non-RD groups and for unilateral kidney segmentation, particularly when segmenting the right kidney from the OP images. Our 3D CNN-assisted automatic segmentation tools can be utilized in future studies on total kidney volume measurements and various image analyses of a large number of patients with CKD.

## Introduction

Chronic kidney disease (CKD) is a common and debilitating disorder that remains a major threat to global public health because of its increasing incidence and mortality. Despite the poor clinical course, most patients with early CKD are unaware of the disease^1^. Therefore, it is important to develop appropriate methods for screening and early diagnosis.

Magnetic resonance imaging (MRI) of the kidneys has been used to noninvasively assess CKD progression. Several MRI methods such as diffusion-weighted imaging (DWI) and blood oxygen level-dependent imaging (BOLD) have been used to evaluate renal function^2–4^. In addition, radiomic analysis using texture features and machine learning algorithms has expanded the scope of MRI. For example, we recently demonstrated the feasibility of texture analysis using kidney MRI for predicting the eGFR in patients with CKD^5^. Although such a quantitative assessment of medical images is more desirable than a qualitative approach, considering inter-rater variability and uncertainty, quantitative methods require segmentation of the areas of interest. This segmentation step can be performed manually; however, it is time-consuming and generates high inter- or intra-rater variability. Therefore, an automatic segmentation approach is required to analyze medical images.

Efforts have been made to develop computerized algorithms to segment various organs. Automatic approaches such as region seed growing^6^, clustering methods, random forests^7^, and deep convolutional neural networks (CNNs)^8^ have been proposed as efficient segmentation tools. Among these, CNN methods are attractive because of their complex hierarchical image recognition techniques, and they attained superior results compared with traditional methods. Several studies have demonstrated that automatic kidney segmentation can be performed using 3D-CNN models. In recent studies, CNN models have achieved excellent results for segmenting kidneys in MRI and CT examinations of patients with adult polycystic kidney disease^9–11^. Guo et al. reported a 3D CNN-based kidney segmentation model with limited training data (≤ 6) using a 3D augmentation process^12^. In a recent study by Daniel et al., CNN-based automatic segmentation was applied to data on normal kidneys and patients with CKD^13^. However, studies on automatic segmentation are limited.

Considering the versatility and generalizability of deep-learning sources, free open-source tools are attractive for facilitating medical image analyses in clinics and research. Several open-source frameworks are available for 3D CNN-based automatic segmentation. Among these, we focused on the new Medical Open Network for Artificial Intelligence (MONAI) framework^14^. MONAI can be easily implemented in an existing deep-learning environment (PyTorch), provides an optimized method to build training workflows, and enables researchers to effectively create state-of-the-art deep-learning models. MONAI contains various deep-learning sources, including 3D CNN-based automatic segmentation tools. To the best of our knowledge, no previous studies have used MONAI 3D CNN-based kidney segmentation.

Thus, in this study, we assessed the feasibility of the MONAI framework for 3D automatic segmentation of the kidney, particularly in patients with CKD, and compared the results with those of previous reports.

## Methods

### Experimental procedure

#### Experiment 1

We used the kidney MRI data of non-renal dysfunction (non-RD) patients. For each participant, we obtained Dixon-based T1 weighted in-phase (IP)/opposed-phase (OP)/water-only (WO) images, and experts manually segmented the entire area of each kidney. We then developed a 3D U-Net-based segmentation model using the training and testing datasets and evaluated the performance in a fivefold cross-validation.

#### Experiment 2

We used kidney MRI data of both renal dysfunction (RD) and non-RD patients. Each kidney of each participant was segmented using Dixon-based T1-weighted IP/OP/WO images. Subsequently, we developed a 3D U-Net-based segmentation model and evaluated its performance using a fivefold cross-validation.

### Subjects

This study was approved by the Research Ethics Committee of the Saitama Medical University Hospital (approval number 2022-107). All experiments were performed in accordance with relevant guidelines and regulations. The requirement for informed consent was waived by the Research Ethics Committee of Saitama Medical University Hospital.

The participants enrolled in this study partially overlapped with those in our previous study on texture analysis using kidney MRI^5^, which was not relevant to the present study. We identified and reviewed 214 patients referred from the Department of Nephrology at our hospital who underwent kidney MRI between January 2017 and December 2021. The inclusion criteria included: (1) age 15 years or older; and (2) MRI scanning with Dixon-based T1-weighted IP/OP/WO images in our hospital. The exclusion criteria included: (1) lack of Dixon-based T1-weighted images (n = 5); (2) insufficient clinical or laboratory data (n = 1); (3) high-grade kidney atrophy (difficulty in segmentation) (n = 2); (4) severe artifacts on MRI (n = 18); and (5) presence of renal lesions with maximal diameter > 1 cm or number of renal masses > 5 in each kidney, including polycystic kidney disease (n = 18). A total of 170 patients were enrolled in this study.

The eGFR was calculated using Eq. (1):

1 $${\text{eGFR }}\left( {{\text{mL}}/{\text{min}}/{1}.{\text{73 m}}^{{2}} } \right) \, = { 194 } \times {\text{ sCr}}^{{ - {1}.0{94}}} \times {\text{ age}}^{{ - 0.{287}}} \times \, 0.{739 }\left( {\text{if female}} \right),$$

where age is in years and serum creatinine (sCr) is in mg/dL. The eGFR was defined as 120 mL/min/1.73 m^2^ if it was greater than 120 mL/min/1.73 m^2^ as calculated using Eq. (1).

The patients were divided into two groups according to the eGFR: 70 patients with RD (eGFR < 45 mL/min/1.73 m^2^, i.e., CKD stage G3b–5) and 100 with non-RD (eGFR ≥ 45 mL/min/1.73 m^2^, i.e., CKD stage G1–3a) groups.

Table 1 details the distribution of the study population in each eGFR group.

**Table 1:** The demographic and clinical characteristics of the study population.

Variable	Non-RD (n = 100)	RD (n = 70)	*P*
Age, years, mean ± SD	51.6 ± 17.4	63.8 ± 14.6	< 0.001
Sex, male	54 (54)	50 (71)	0.02
Hypertension	33 (33)	47 (67)	< 0.001
Diabetes	7 (7)	19 (27)	< 0.001
IgA nephropathy	14 (14)	9 (13)	0.83
Nephrotic syndrome	4 (4)	1 (1)	0.33
eGFR, mL/min/1.73 m^2^, mean ± SD	64.2 ± 17.6	29.1 ± 11	< 0.001

Except where otherwise indicated, data are presented as number (%) of patients.

*RD* renal dysfunction (eGFR < 45 mL/min/1.73 m^2^, i.e., CKD stage G3b-5), *non-RD* non-renal dysfunction (eGFR ≥ 45 mL/min/1.73 m^2^, i.e., CKD stage G1-3a), *IgA* immunoglobulin A, *SD* standard deviation.

### Data

As described above, we prepared two datasets for the kidney MRI of the patients for the two experiments. The dataset for Experiment 1 contained 100 kidney MRI scans from 100 non-RD patients. The second dataset for Experiment 2 contained 170 kidney MRI scans from 70 patients with RD and 100 patients without RD. For all patients in these datasets, we obtained Dixon-based T1-weighted IP/OP/WO images (only IP/OP/WO images were used in the analysis because other images, such as fat-only images and fat fraction ratio maps, were not generated for all patients). Each image volume contained 40–48 2D coronal slices and each slice was 320 × 320 pixels in size. The coronal image slice spacing was 3.0 mm and the in-plane pixel resolution was 1.125 × 1.125 mm^2^.

MRI images were acquired using a 3.0 Tesla superconducting unit (Skyra; Siemens Healthcare, Erlangen, Germany) with a spine coil and an 18-channel phased-array body coil. Representative scanning parameters for T1-weighted IP/OP/WO images were as follows: repetition time = 5.35 ms, echo time = 2.46 and 3.69 ms, flip angle = 10°, slice thickness = 3 mm, field of view = 360 × 360 × 144 mm, and recon matrix = 320.

The kidney segmentation for all these datasets was drawn manually by two expert radiologists with 7 and 8 years of experience (K.N. and Y.H., both with 2 years of experience performing image segmentation/annotation in other studies) using open-source software (ITK-SNAP version 3.8.0). One radiologist (K.N.) first delineated the region of interest (ROI), and a second radiologist (Y.H.) confirmed and corrected the segmentation, if needed.

Our final dataset included renal lesions ≤ 1 cm in maximum diameter and ≤ 5 in number: 23 cases for RD group and 19 cases for non-RD group. All these lesions were renal cysts and not renal tumors. During the manual labeling by radiologists, these lesions were omitted from the masks.

### Image processing and model implementation

We randomly split the data into 70% for training, 10% for validation, and 20% for testing (i.e., 70, 10, and 20 patients for Experiment 1, and 119, 17, and 34 patients for Experiment 2, respectively). We performed a fivefold cross-validation, each fold with 20% of the available data for the test. This enabled the models to be tested on the entire dataset. The auto-segmentation model was trained using Python 3.9 (Python Software Foundation, Beaverton, OR) and the MONAI library (v1.1.0, https://monai.io/) with Pytorch 1.9.0 (Facebook’s AI Research Laboratory) backend. All DICOM image data were converted to the Neuroimaging Informatics Technology Initiative (NifTI) format. Data transformation and augmentation were performed using MONAI transformations: the 3D orientation method performs right to left, anterior to posterior, and superior to inferior spacing to resample the input image into a specified output voxel spacing; normalizes the voxel intensity distribution of each image by rescaling the intensities into the range of [0, 255]; rescales the intensities between 0 and 1 and removes all zero areas to focus on the valid body area of the images and labels; randomly crops patch samples from large images based on positive/negative ratios; and uses a random affine, which performs rotation, scaling, and translation in a fixed-sized region. We used the MONAI “CacheDataset” tool to load the pre-processed data. This dataset loader accelerates the training and validation processes. Details of the model implementation are described in Supplementary Information.

**Figure 1:**
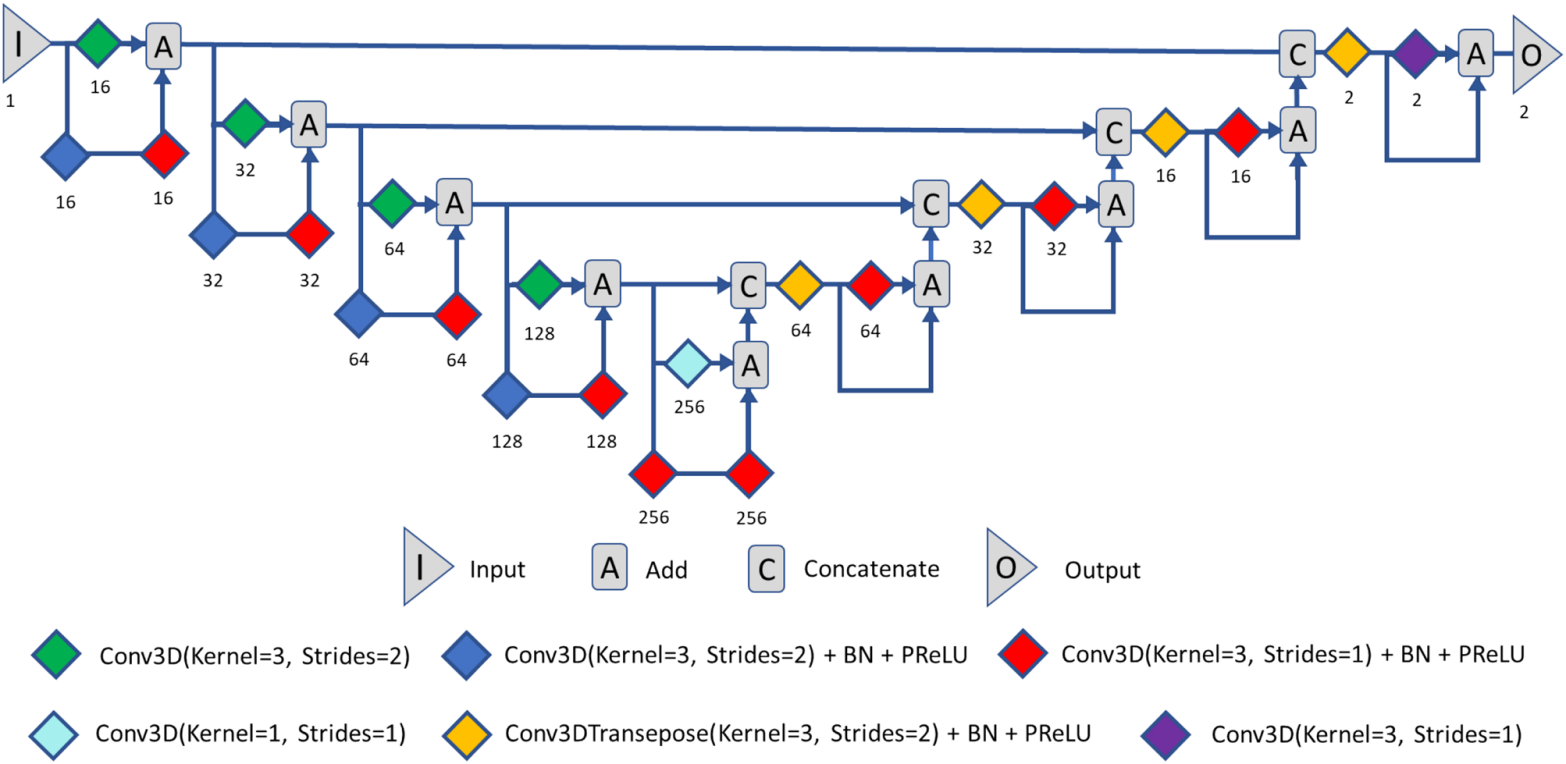
An overview of our three-dimensional convolutional neural network (3D CNN) model used for kidney segmentation. Input is a T1-weighted kidney MRI, followed by 3D convolutional (Conv3D) layers with four convolution blocks in the encoding and decoding branches and a bottleneck convolution block between the two branches. The number of channels is given above each block. The batch normalization (BN) and the parametric rectified linear unit (PReLU) layers are indicated.

Our segmentation model was based on a 3D U-Net architecture with residual connections (ResUNet) included in the MONAI software package. Figure 1 shows the 3D CNN model used for kidney segmentation. The details of our architecture are described in the Supplementary Information. We used the Adam optimizer to compute the parameter updates and the Dice loss function. The values for the Adam optimizer coefficients b1 and b2 were 0.9 and 0.999, respectively. The models were trained for 500 iterations on a Windows 10 workstation with a single GeForce RTX 3090 Graphics Processing Unit. Overall, five models were trained (one per fold), with a training time of approximately 3–4 h per fold. Using the trained model to perform automatic kidney segmentation from the test data required 4–5 s per case. Finally, the model predictions were averaged and compared with manual segmentations using the MONAI mean Dice, 95% Hausdorff distance, and average surface distance metrics. The mean volume of the predicted kidney masks was also obtained, as was the mean volume difference between the predicted and ground-truth masks (i.e., the CNN-predicted volume − ground-truth volume).

Statistical analyses were performed using the open-source software package (Python scikit-learn 0.22.1). Statistical significance was set at P < 0.05.

## Results

### Experiment 1

**Figure 2:**
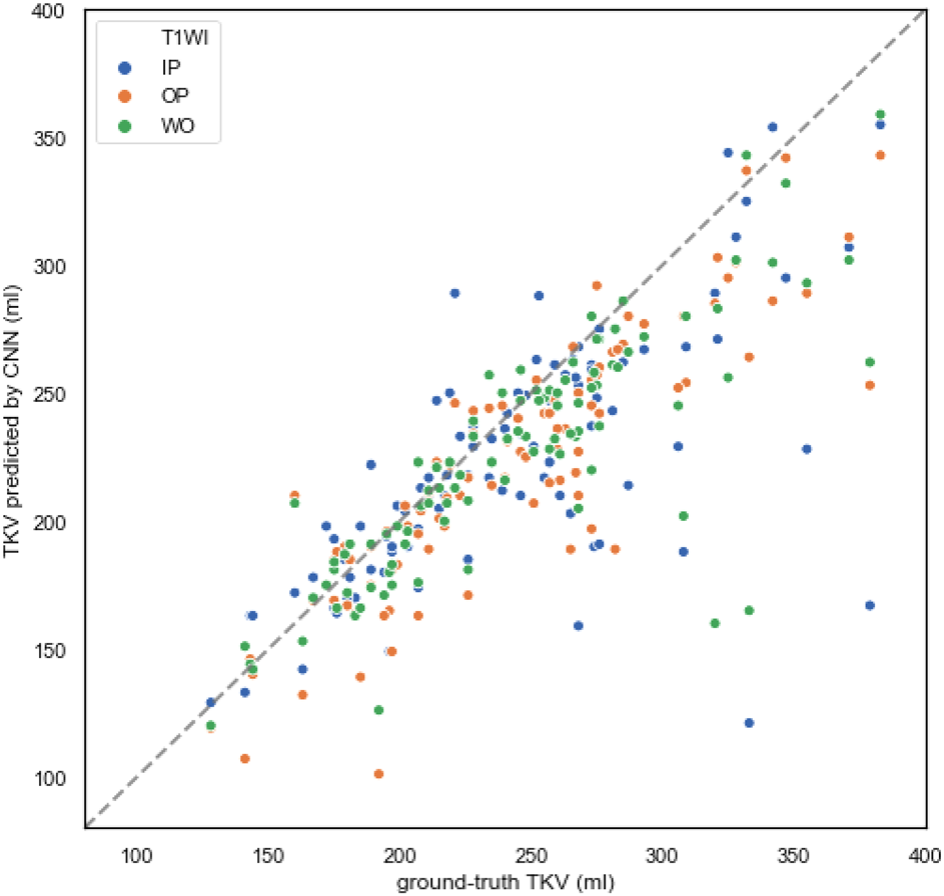
The scatter plot of the total kidney volume (TKV) predicted by convolutional neural network (CNN) against the ground-truth TKV in non-renal dysfunction (non-RD) cases with T1-weighted in-phase, opposed-phase, and water-only image (T1WI IP/OP/WO) denoted by blue, orange, and green dots, respectively. The dotted line represents perfect correlation between the CNN-predicted and ground-truth segmentation.

The best performance was obtained when using IP image, with a Dice score of 0.902 ± 0.034, average surface distance of 1.46 ± 0.75 mm, and volume difference of − 27 ± 21 mL. A summary of the CNN-predicted segmentation accuracy evaluated using similarity metrics and the volume difference from the ground-truth segmentation is presented in Table 2. Figure 2 shows plots of the correlation between the ground-truth total kidney volume (TKV) and the TKV predicted by the CNN.

**Table 2:** The performance of CNN-predicted kidney segmentation in non-RD cases (Experiment 1).

	Total			Left			Right		
	IP	OP	WO	IP	OP	WO	IP	OP	WO
Dice score	0.902 ± 0.034	0.880 ± 0.060	0.892 ± 0.036	0.887 ± 0.022	0.877 ± 0.044	0.871 ± 0.050	0.886 ± 0.079	0.848 ± 0.103	0.910 ± 0.010
Hausdorff distance (mm) (95th percentile)	5.91 ± 8.34	22.97 ± 19.09	28.33 ± 23.20	32.31 ± 37.81	57.02 ± 29.44	72.96 ± 41.81	72.62 ± 59.02	71.71 ± 55.87	41.51 ± 43.08
Average surface distance (mm)	1.46 ± 0.75	3.17 ± 2.11	3.91 ± 2.23	5.66 ± 4.45	9.58 ± 6.67	12.38 ± 6.53	11.91 ± 14.14	17.26 ± 17.84	7.00 ± 5.58
Volume (mL)	222 ± 8	221 ± 12	224 ± 10	107 ± 5	103 ± 5	116 ± 5	113 ± 9	106 ± 7	112 ± 7
Volume difference	− 27 ± 21	− 27 ± 20	− 25 ± 19	− 19 ± 11	− 23 ± 11	− 10 ± 10	− 10 ± 10	− 17 ± 9	− 11 ± 14

Data are presented as means ± standard deviation.

*non-RD* non-renal dysfunction (eGFR ≥ 45 mL/min/1.73 m^2^, i.e., CKD stage G1-3a), *IP* in-phase, *OP* opposed-phase, *WO* water-only.

**Figure 3:**
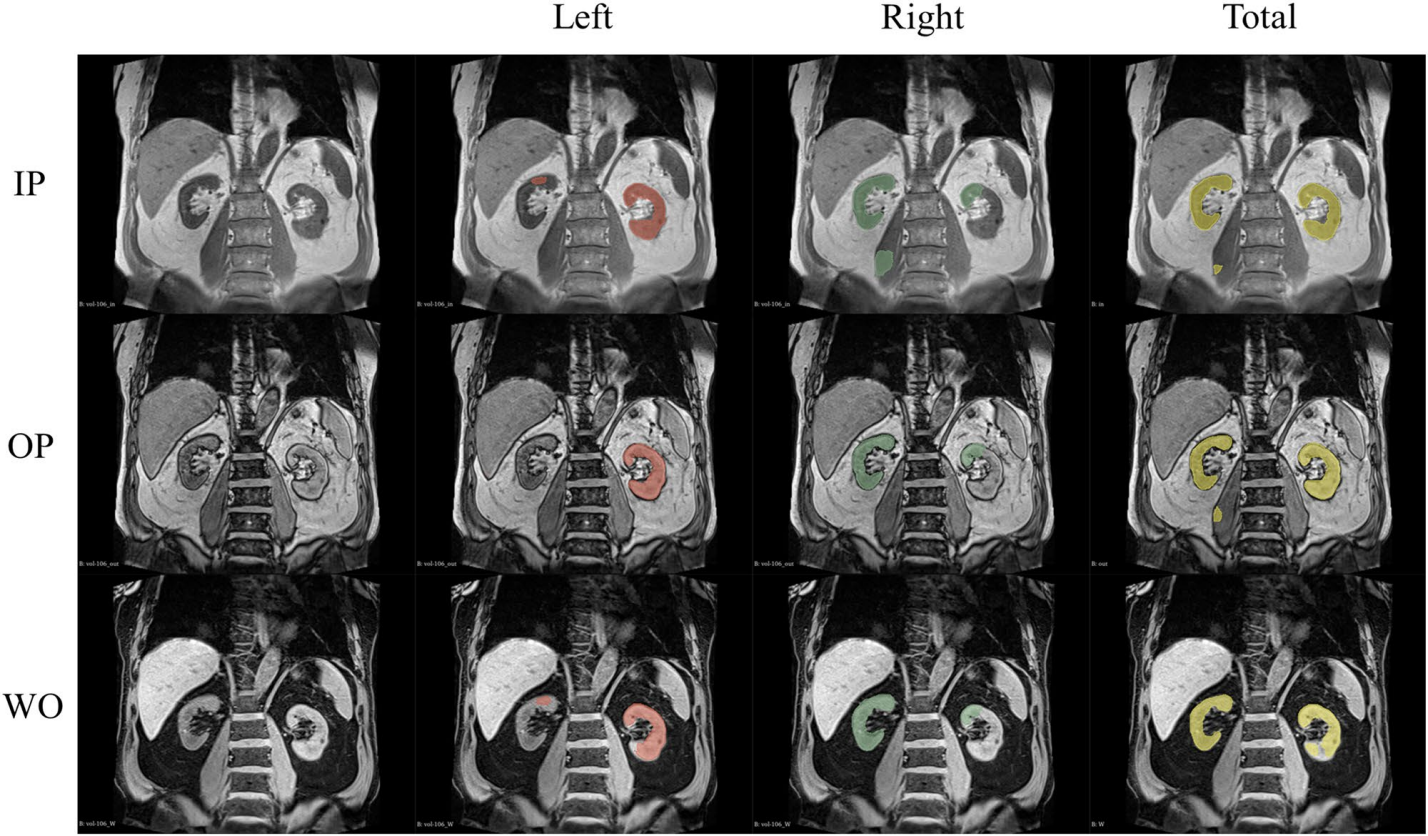
An example of test images and corresponding convolutional neural network (CNN)-predicted masks of a non-renal dysfunction (RD) patient. From top to bottom: T1-weighted in-phase (IP), opposed-phase (OP), and water-only (WO) images. From left to right: raw image data, and the masks of the left kidney (red), right kidney (green), and both kidneys (yellow). Note that over-segmentation of the medial portion of the superior pole of the contralateral kidney was frequently observed in the unilateral kidney segmentation. Another frequent mis-segmentation occurred in the psoas major adjacent to the kidney.

Figure 3 illustrates an example of a mask produced by a CNN. Overall, poor segmentation was observed in the ventral, dorsal, and upper pole portions of the kidney. Oversegmentation of the psoas major, spleen, liver, aorta, duodenum, and small intestine was observed. An incorrect segmentation of the medial upper portion of the contralateral kidney is frequently observed in unilateral kidney segmentation.

### Experiment 2

**Figure 4:**
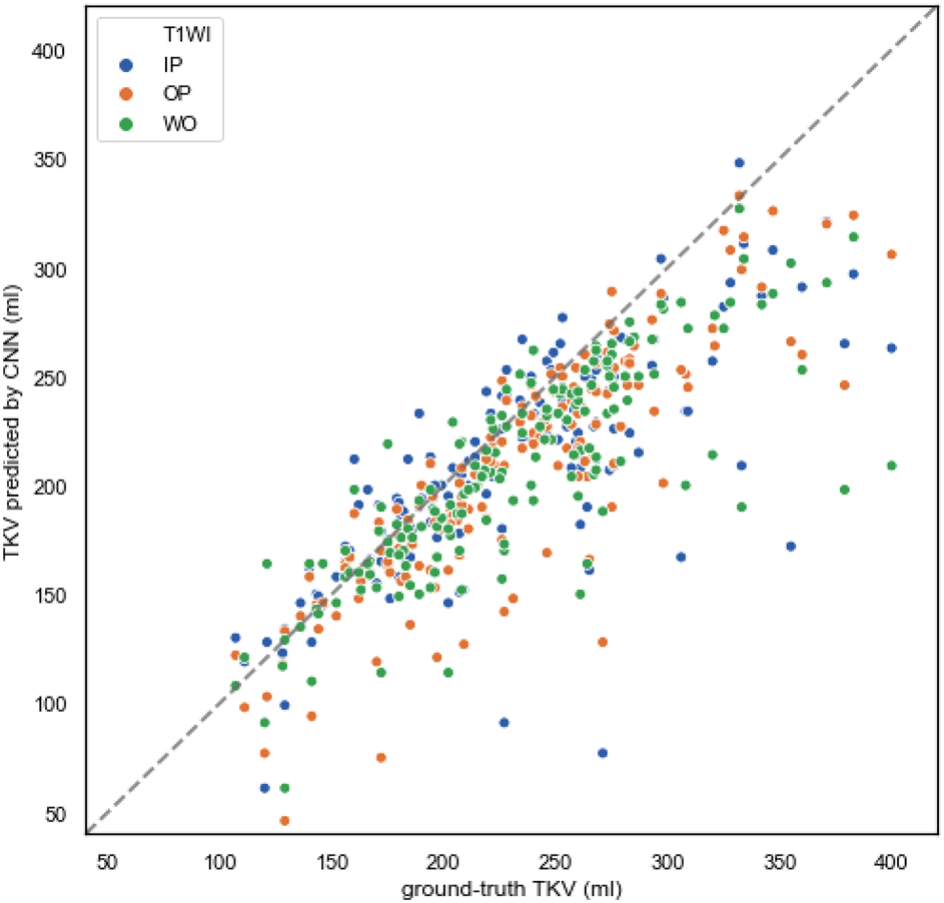
The scatter plot of the total kidney volume (TKV) predicted by convolutional neural network (CNN) against the ground-truth TKV in renal dysfunction (RD) and non-RD cases with T1-weighted in-phase, opposed-phase, and water-only image (T1WI IP/OP/WO) denoted by blue, orange, and green dots, respectively. The dotted line represents perfect correlation between the CNN-predicted and ground-truth segmentation.

A good performance was obtained when using OP image, with a Dice score of 0.894 ± 0.035, average surface distance of 2.22 ± 1.18 mm, and volume difference of − 32 ± 16 mL. A summary of the CNN-predicted segmentation accuracy when evaluated using similarity metrics and the volume difference from the ground-truth segmentation is presented in Table 3. Figure 4 shows plots of the correlation between the ground-truth TKV and the TKV predicted by the CNN.

**Table 3:** The performance of CNN-predicted kidney segmentation in RD and non-RD cases (Experiment 2).

	Total			Left			Right		
	IP	OP	WO	IP	OP	WO	IP	OP	WO
Dice score	0.887 ± 0.031	0.894 ± 0.035	0.893 ± 0.039	0.869 ± 0.031	0.863 ± 0.048	0.864 ± 0.070	0.865 ± 0.117	0.799 ± 0.122	0.780 ± 0.129
Hausdorff distance (mm) (95th percentile)	20.75 ± 24.58	13.41 ± 14.02	36.11 ± 29.74	46.16 ± 36.07	60.51 ± 43.77	45.98 ± 31.15	36.87 ± 44.27	55.90 ± 46.19	82.68 ± 17.54
Average surface distance (mm)	2.79 ± 3.25	2.22 ± 1.18	4.73 ± 3.83	8.74 ± 6.50	10.75 ± 7.53	8.73 ± 5.32	9.41 ± 11.18	21.55 ± 17.17	24.18 ± 14.26
Volume (mL)	209 ± 11	205 ± 16	207 ± 7	104 ± 5	108 ± 8	113 ± 19	104 ± 7	102 ± 5	105 ± 4
Volume difference	− 28 ± 16	− 32 ± 16	− 30 ± 16	− 16 ± 10	− 12 ± 9	− 7 ± 11	− 13 ± 4	− 16 ± 8	− 12 ± 6

Data are presented as means ± standard deviation.

*RD* renal dysfunction (eGFR < 45 mL/min/1.73 m^2^, i.e., CKD stage G3b-5), *non-RD* non-renal dysfunction (eGFR ≥ 45 mL/min/1.73 m^2^, i.e., CKD stage G1-3a), *IP* in-phase, *OP* opposed-phase, *WO* water-only.

Overall, the trend of oversegmentation was the same as that described above; however, the incorrectly segmented areas in the medial renal region tended to be wider than those in Experiment 1.

## Discussion

In this study, we investigated the feasibility of 3D CNN-based automatic kidney segmentation by using T1-weighted IP/OP/WO MRIs. The results of this study suggest that the 3D ResUNet architecture of the MONAI framework can successfully segment kidneys. The overall accuracy was good for bilateral kidney segmentation, either in non-RD-only kidneys or both RD and non-RD kidneys. Slightly worse results were obtained for unilateral kidney segmentation and for combined RD and non-RD kidneys than for non-RD kidneys alone, especially when segmenting the right kidney and OP images.

CNN-based automatic kidney segmentation methods have been studied previously. Some studies have focused on segmentation of polycystic kidney disease^9–11^. The 3D CNN model exhibited excellent performance in segmenting the kidney in MRI T2-weighted images of adult patients with polycystic kidney disease^10^. However, to build such a model required 2000 image and segment datasets for training and another 400 datasets for testing.

A 3D CNN-based kidney segmentation method with limited training data using a data augmentation process was examined. Guo et al. investigated a 3D augmentation and cascaded CNN approach using a small number of subjects for training and testing^12^. Their approach yielded mean Dice coefficients of 0.85 using a single training subject and 0.91 with six training subjects. Although our results are comparable to theirs, it is noteworthy that they achieved high performance using only a few training subjects. One limitation they mentioned was that their approach required a training time of up to two weeks using a single GPU with 32 GB (Nvidia v100 GPU)^12^. However, in actual use, manual data preparation requires comparable time (approximately 2 weeks for 100 cases or more in our study), so it is not easy to judge which method is more time-saving.

In another study, Daniel et al. recently reported 2D CNN-based kidney segmentation from T2-weighted MRI to calculate the TKV in healthy controls and patients with CKD^13^. The differences between their study and ours were the smaller sample size (30 healthy controls and 30 patients with CKD) and 2D CNN-based approach. They yielded fairly good performances, with a mean Dice score of 0.94 ± 0.01 and 0.93 ± 0.01, average surface distance of 0.68 ± 0.27 and 0.65 ± 0.21, and volume difference of 4.66 ± 17.72 and − 1.16 ± 16.23, for healthy control group only and healthy control and CKD group, respectively^13^. However, our results were worse than theirs. In particular, the volume difference was evidently negative (a CNN-based volume smaller than the ground-truth volume), which implies undersegmentation caused by poor segmentation of the ventral, dorsal, and upper pole portions of the kidney. In contrast, in their work, there were no major inconsistencies in kidney volumes between the manual and CNN-based approaches, except for a lower accuracy in defining the kidney-spleen boundaries^13^. At the same time, the average surface and Hausdorf distances were higher in our study than in theirs, which could be caused by the oversegmentation of other unrelated regions, such as the psoas major, spleen, liver, aorta, duodenum, and small intestine. Furthermore, this study found large variations in the overall results, especially in the average surface and Hausdorff distances, which could also be explained by oversegmentation. Because the oversegmented regions described above were either near or far from the kidney, variations may be reflected in distance rather than in volume.

Our study used the 3D ResUNet architecture for the automated segmentation of the kidney. In the study by Daniel et al., a 2D CNN was used instead, in which each 2D slice of the full-volume data was processed separately^13^. The 2D CNN approach is advantageous for relatively small datasets because it avoids overfitting and allows the network to be easily used on volumes with a variable number of slices. However, 2D CNNs are considered less accurate because they cannot take advantage of neighboring slice information in their computations. In contrast, 3D CNNs have disadvantages in terms of computational cost and time required; however, they have been applied in several recent studies on automated segmentation approaches with successful results. In 3D networks, the image volume is divided into smaller cubes to allow different input shapes, thus reducing memory requirements^15^. Our study demonstrated that the 3D ResUNet framework of MONAI can be successfully applied to automatic kidney segmentation. In the MONAI libraries, a tool to load the pre-processed data “CacheDataset” could be used to allow acceleration of training and validation^14^. Using this tool, we completed the training and validation processes in approximately 3–4 h, which is comparable to that of a previous 2D CNN-based study^13^.

Our study showed that the automated segmentation of both kidneys based on T1-weighted IP, OP, or WO images could be performed with sufficient accuracy. We observed a smaller CNN-based TKV than the ground-truth TKV, which was mostly due to poor segmentation of the ventral, dorsal, and upper pole portions of the kidney. On coronal images, the ventral portion of the kidney was surrounded by the gastrointestinal tract and the dorsal portion was proximal to the psoas major. The upper pole portion of the kidney is adjacent to the liver on the right, the spleen on the left, and other structures such as the adrenal glands, blood vessels, and vertebral bodies. This may make it difficult to recognize kidneys using a CNN.

A unique feature of the kidneys is that they are paired organs. Segmentation of both kidneys might serve most purposes; however, when assessing the left or right kidneys separately, the masks of both kidneys must be divided into two parts, which is cumbersome. Furthermore, separate autosegmentation of the right and left kidneys may be meaningful because it can help identify how the CNN distinguishes between the two.

Several factors were considered for the CNN to distinguish between right and left kidneys. One factor might be the position of the kidney relative to the adjacent liver and spleen and the contrast between the kidney and these organs. In our study, adjacent parenchymal organs served as references when the CNN discriminated between the left and right renal parenchyma and in areas surrounded by adipose tissue, as it was difficult to identify the left and right kidneys because there were no surrounding organs. Therefore, in this study, when there was a large amount of fat in the retroperitoneal region, the segmentation accuracy tended to be worse than when there was less fat.

The contrast between the kidneys and adjacent organs is another point of consideration. A previous report using T2-weighted images for automatic kidney segmentation demonstrated that the segmentation accuracy was better for the left than for the right kidney^13^, and it was suggested that the proximity and lack of contrast between the left kidney and spleen made distinguishing this boundary difficult for the CNN. On the Dixon-based T1-weighted images used in our analysis, the overall signal intensity of the kidney was lower than that of the liver, and similar to that of the spleen. Importantly, the effects of a fatty liver must be considered. Owing to the deposition of fat in the liver, the signal intensity of the liver is typically reduced in OP images, but unchanged in IP or WO images. Therefore, in fatty liver, the contrast between the right kidney and the liver could decrease in the OP images. However, in our study, renal segmentation was successfully performed in many cases of fatty liver. Thus, it seems that the change in contrast due to fatty liver did not pose much of a problem in the identification of the right kidney.

Another factor enabling the CNN to distinguish between the right and left kidneys could be the slight difference in the signal intensity patterns of the left and right kidneys on Dixon-based T1-weighted images. This is one of the strengths of neural networks; however, such differences cannot be easily perceived by the human eye. The incorrect segmentation frequently observed in this study can be explained from this perspective. In this study, there seemed to be a similarity in the depiction, for example, between the kidney and the ipsilateral psoas major, which might have led to the segmentation of the psoas major. Furthermore, our CNN-based unilateral kidney segmentation method often oversegmented the medial portion of the superior pole of the contralateral kidney, which could be due to the similarity in the signal intensity patterns on both sides of the medial upper part of the kidney.

In this study, the agreement between the CNN-based and ground-truth masks was higher for the left kidney than for the right kidney. This result contrasts with that of a previous study that used T2-weighted images for automatic kidney segmentation^13^ and could be caused by the factors described above. Incorrect segmentation of the medial upper portion of the contralateral kidney was more frequently observed in the right kidney than in the left. This may be because the adjacent fat region and the aforementioned area of signal intensity similarity were wider in this part of the left kidney than in the right kidney. In addition, the adjacent psoas major muscle could be partially oversegmented in the segmentation of the right kidney, which might also be due to the similarity in signal intensity and pattern between the right psoas muscle and kidney.

This study explored the feasibility of the automatic segmentation of both RD and non-RD kidneys. The general trend of mis-segmentation in RD patients was the same as that in non-RD patients; however, oversegmented areas in the medial renal region tended to be wider than in non-RD patients. A relative increase in fat in the retroperitoneal region due to renal atrophy in the RD could lead to the absence of neighboring organs that serve as hints and exacerbate segmentation accuracy. Furthermore, irregular deformation or atrophy and reduced corticomedullary contrast can result in poor segmentation accuracy. However, inferior results were observed in unilateral kidney segmentation. Good segmentation accuracy was confirmed in the segmentation of both kidneys, which was comparable to that in a previous report^13^.

Our study had several limitations. First, we retrospectively enrolled 170 patients from a single institution, which had a small sample size with an imbalance between the RD and non-RD groups. A larger number of patients with more balanced groupings is needed to validate these results. Second, because we excluded patients with renal lesions, some important renal diseases such as polycystic kidney disease were ignored in this analysis, which could have caused selection bias. Third, because the data were analyzed using the internal cross-validation method because of the limited number of patients, further investigations using an independent external validation cohort should be performed. Finally, we could not analyze other Dixon-based images such as FO images and fat fraction ratio maps because they were not available for all patients.

Furthermore, T2-weighted images could not be used in this study because they were scanned in the axial or coronal planes in our routine sequence. In a recent report, T2-weighted images provided better quality and reproducibility for TKV assessment than T1-weighted images in patients with polycystic kidney disease^16^. Therefore, segmentation using these sequences should be examined in the future to compare their performance.

In conclusion, we developed a 3D CNN-based automatic kidney segmentation method for patients with CKD using MRI Dixon-based T1-weighted images. The overall accuracy was good for bilateral kidney segmentation. Slightly worse results were obtained for unilateral kidney segmentation, and the results were worse in both groups than in the non-RD kidneys only, particularly when segmenting the right kidney and OP images. These computer-assisted segmentation tools may be used in future studies on TKV measurements and image analyses in a large number of patients with CKD.

### Supplementary Information

Supplementary Information.

## Data Availability

The authors declare that all the data supporting the findings of this study are available within the article.

## Abbreviations

BOLD: Blood oxygen level-dependent imaging
CKD: Chronic kidney disease
CNN: Convolutional neural network
DWI: Diffusion-weighted imaging
eGFR: Estimated glomerular filtration rate
IP: In-phase
Non-RD: Non-renal dysfunction
MONAI: Medical Open Network for Artificial Intelligence
MRI: Magnetic resonance imaging
OP: Opposed-phase
RD: Renal dysfunction
ROI: Region of interest
TKV: Total kidney volume
WO: Water-only
