# Supplementary material for: The utility of automatic segmentation of kidney MRI in chronic kidney disease using a 3D convolutional neural network
Source: Sci Rep. 2023 Oct 13;13:17361. doi: 10.1038/s41598-023-44539-z (PMC10575938; doi:10.1038/s41598-023-44539-z)

**Supplementary Information**

The processed MR images and kidney masks were randomly cropped into four fixed-size regions (patches) (64, 64, 64) per patch per patient. Random spatial cropping considered the foreground (i.e., the kidney section) and background (i.e., the non-kidney section) in the sampling, with a 50% probability for both kidney and non-kidney sections. For a batch of training examples, we used two patient images, and therefore, eight patches of images. The shape of the input tensor provided to the network was (8, 1, 64, 64, 64) for an effective batch size of 8, one-channel input (MRI), and patch size of (64, 64, 64).

**Auto-segmentation model**


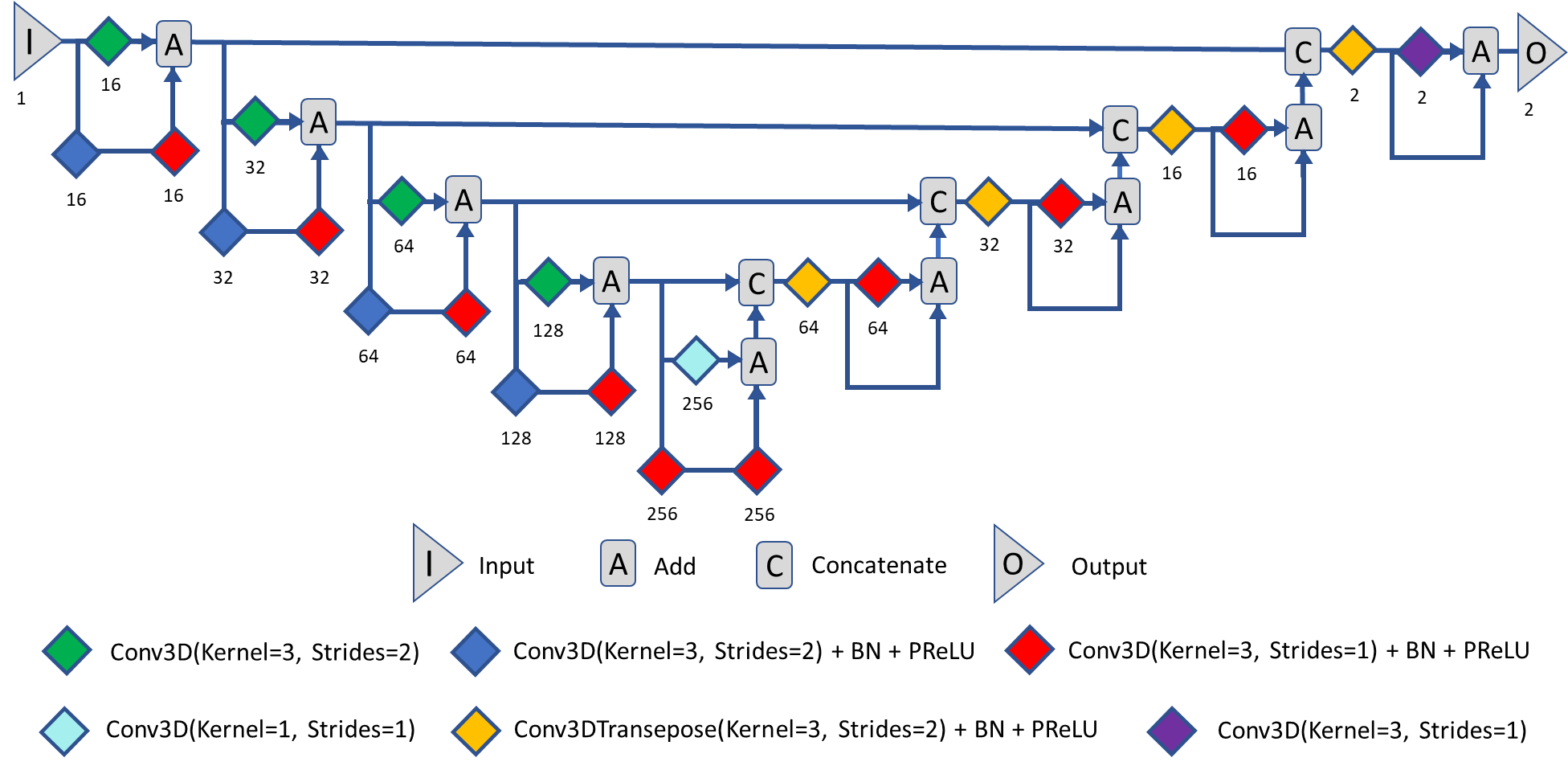


The 3D ResUNet architecture is illustrated in the above figure. Overall, it consists of four convolution blocks in the encoding and decoding branches and a bottleneck convolution block between the two branches. All the convolution layers used a kernel size of three, except for one convolution layer in the bottleneck, which used a kernel size of one. The numbers of channels in the convolution layers were 16, 32, 64, 128, and 256. Each convolution block in the encoding branch comprised a two-strided convolution layer, a residual connection containing a two-strided convolution layer, and a one-strided convolution layer. In the bottleneck, the residual connection contains two one-stride convolutional layers. In the decoding branch, each block contains a two-stride transpose convolution layer, a one-stride convolution layer, and a residual connection. Batch normalization and parametric rectified linear unit activation functions are used throughout the architecture. An MRI image was used as the one-channel input for the model. The kidney segmentation mask was provided as a two-channel output in one-hot encoding, with the first channel being the probability map of the background, and the second channel being the region of interest.

**Source code:**


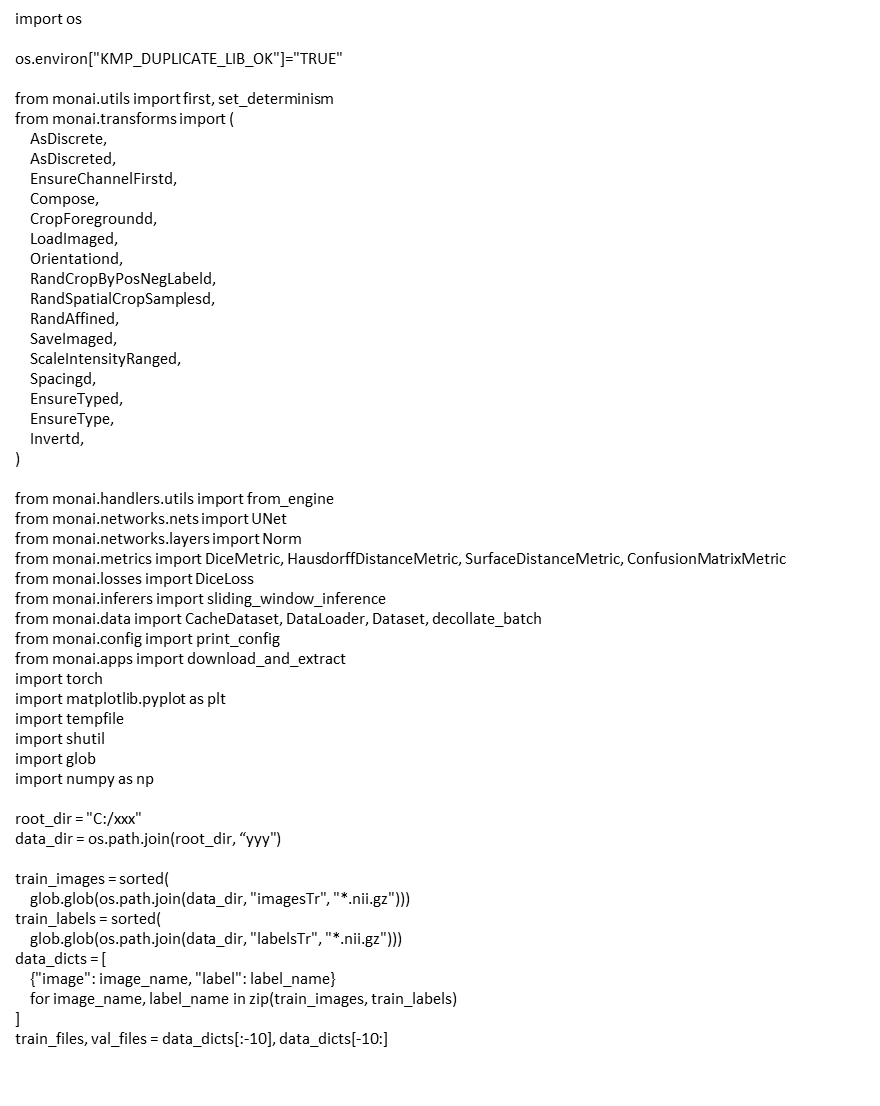


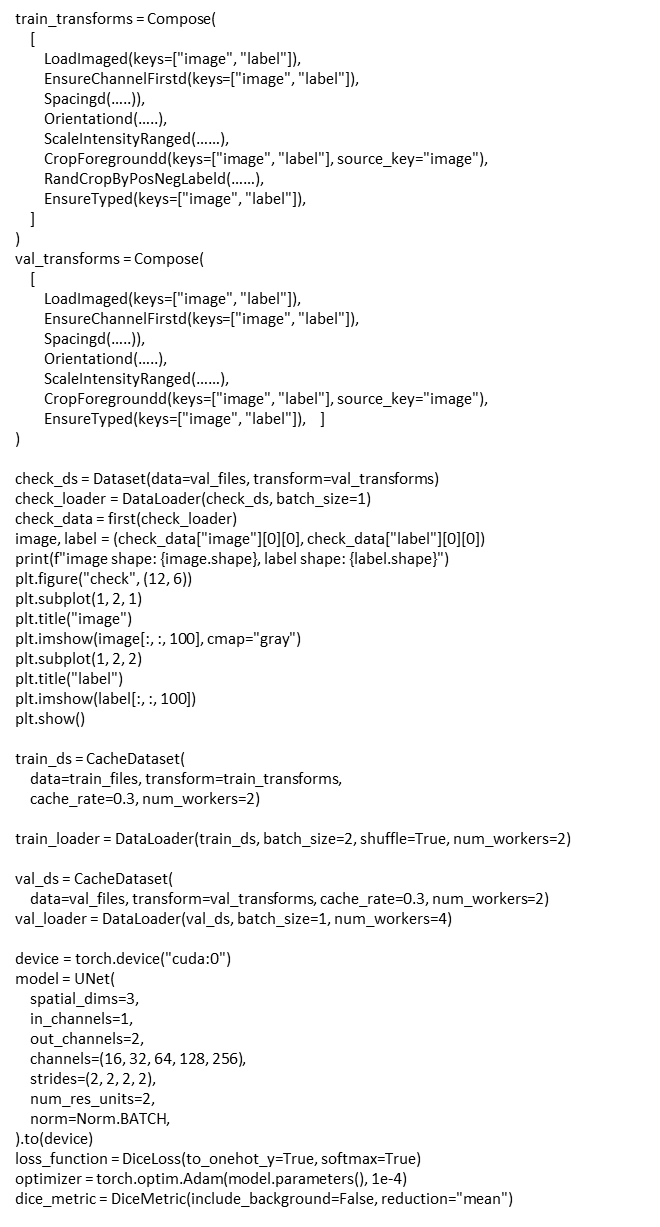


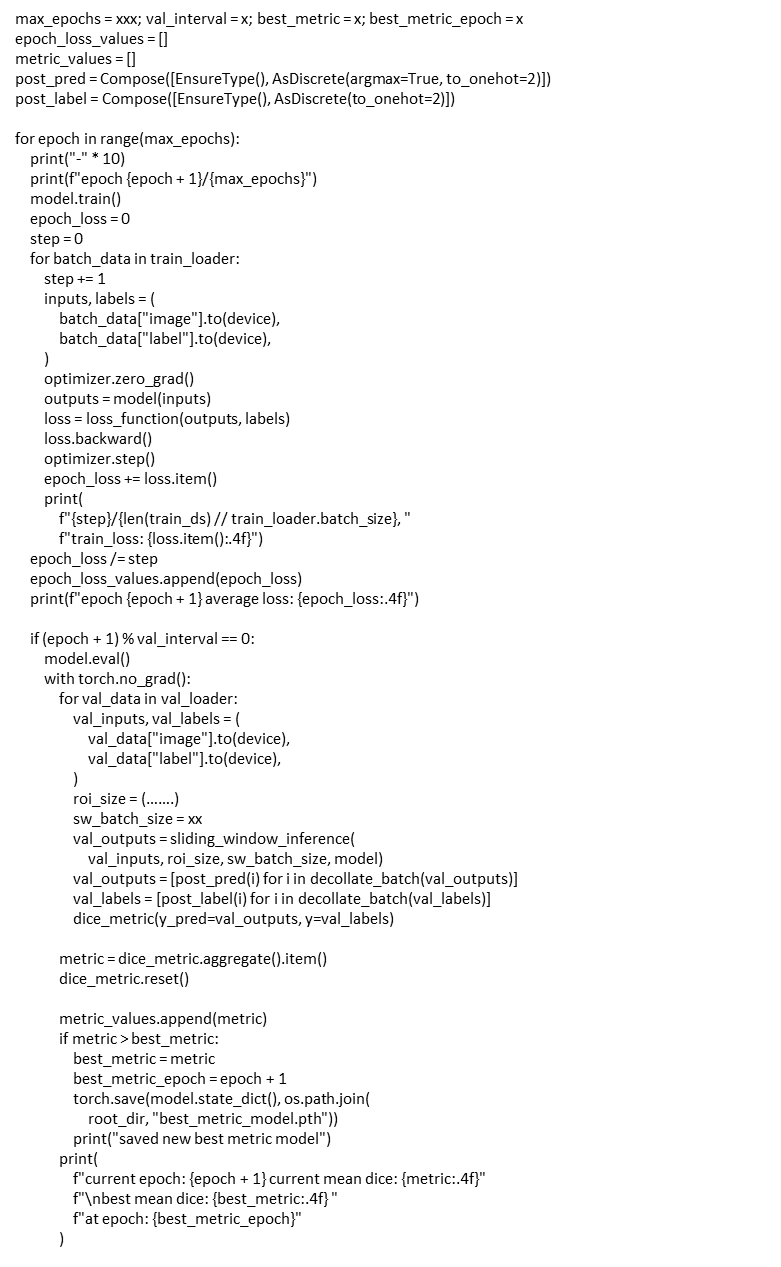


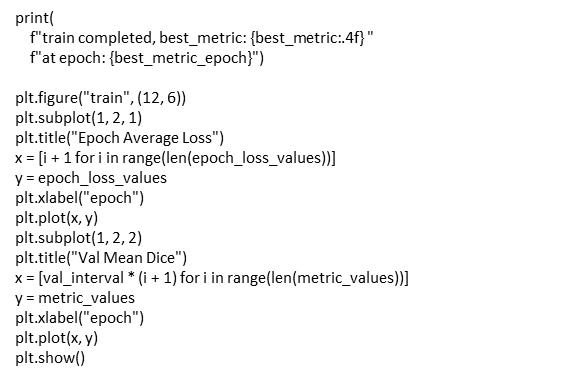

Supplement: Supplementary file 1 — Supplementary Information. [file 41598_2023_44539_MOESM1_ESM.docx]
